# Network-constrained Random Lasso for biologically interpretable gene network inference across unequal sample sizes

**DOI:** 10.1371/journal.pone.0344198

**Published:** 2026-03-31

**Authors:** Heewon Park, Satoru Miyano

**Affiliations:** 1 School of Mathematics, Statistics and Data Science, Sungshin Women’s University, Seoul, Republic of Korea; 2 M&D Data Science Center, Tokyo Medical and Dental University, Bunkyo-ku, Tokyo, Japan; 3 Human Genome Center, The Institute of Medical Science, The University of Tokyo, Minato-ku, Tokyo, Japan; University of Idaho, UNITED STATES OF AMERICA

## Abstract

Gene regulatory network inference is a key approach for elucidating molecular mechanisms underlying complex diseases, but accurately inferring them from high-dimensional data, especially when sample sizes are imbalanced, remains a significant challenge. Although the *L*_1_-type regularization methods have been used for gene network inference, the existing methods often fail under conditions involving high dimensionality, noise, and unequal sample sizes across phenotypes. To overcome these limitations, this study developed netRL, a novel computational framework that integrates the Random Lasso with prior network biological knowledge. The proposed method leveraged a bootstrap-based strategy to stabilize the selection of key regulatory genes and incorporates network-informed penalization using centrality measures (i.e., hubness and betweenness centrality). This study also introduced a statistical strategy using a hypergeometric test to assess the significance of the inferred edges, thereby enhancing the reliability of the network. Through extensive simulation studies, this study demonstrated that netRL outperforms conventional methods in both network estimation and gene selection. Applying netRL to whole-blood RNA-seq profiles from the Japan COVID-19 Task Force, this study successfully identified distinct phenotype-specific molecular interplays between asymptomatic and critical cases despite pronounced sample imbalance. The findings reveal that asymptomatic networks were dense and enriched for ribosomal proteins, whereas critical networks were sparse, centralized, and characterized by hub genes such as NFKBIA, B2M, CXCL8, and FOS. Pathway enrichment further revealed phenotype-specific biological processes, highlighting molecular signatures of disease progression. The results of this study suggest that enhancing the activity of asymptomatic condition-specific markers (e.g., ribosomal proteins) may provide important insights into the molecular mechanisms underlying COVID-19 severity. Collectively, these results demonstrate that netRL enables biologically interpretable and statistically robust network inference, offering new insights into the molecular basis of COVID-19 severity and broader applications in systems biology.

## Introduction

Gene regulatory networks (GRNs) provide a powerful framework to represent molecular interactions, where nodes correspond to genes and edges denote regulatory influences. Accurately reconstructing GRNs from high-dimensional transcriptomic data enables researchers to elucidate disease mechanisms, identify biomarkers, and uncover therapeutic targets. With the increasing availability of large-scale RNA sequencing data, the development of statistically rigorous and biologically interpretable computational methods for network inference has become essential.

However, a significant challenge in gene network inference, particularly in clinical and biological studies, is the issue of imbalanced sample sizes across different phenotypes or conditions. For instance, in rare diseases or specific disease subtypes, the number of available samples for one group may be substantially smaller than for a control group. This disparity can lead to several statistical problems, including estimation bias and a significant reduction in statistical power. As a result, the molecular interactions identified in such studies are more likely to be artifacts of the sampling imbalance rather than those stemming from true biological differences. Although various *L*_1_-type regularization methods (e.g., lasso, adaptive lasso, and elastic net) have been developed and widely applied in gene network inference, most existing computational methods are highly susceptible to the aforementioned issues. Furthermore, the lasso method tends to select only a portion of correlated genes, potentially overlooking biologically relevant groups. Although the elastic net can circumvent some of these limitations, it may produce biased network estimates when correlated genes have edges that differ in magnitude of weight or direction to their target genes, which frequently occurs in gene networks [2]. While some methods have been developed to address these limitations, they often overlook crucial biological context, leading to networks that are statistically sound but lack biological interpretability. For example, although the standard Random Lasso framework [2] offers advantages in feature selection, it lacks integration of biological knowledge, which can be critical for accurately inferring biologically consequential networks.

To address these challenges, this study developed network-constrained Random Lasso (netRL), a novel framework that integrates random lasso with network biological knowledge. The developed netRL leverages a bootstrap-based strategy to mitigate sample imbalance while enhancing the stability of regulator genes selection. The proposed strategy measure the gene importance based on the not only statistical but also network biological knowledge (i.e., hubness and between centrality). In addition, netRL incorporates prior molecular interaction knowledge into the penalty structure, thereby steering genes selection toward biologically plausible solutions. To further refine inference, this study developed statistical strategies that assesses the significance of estimated edges within the bootstrap framework using the hypergeometric test. By combining these complementary components—bootstrap-based stabilization, network-informed penalization, and statistical edge validation—netRL offers a robust and biologically interpretable solution for gene network inference. The framework enables accurate detection of phenotype-specific molecular interplays under challenging conditions such as high dimensionality, noise, and unequal sample sizes, ultimately facilitating the discovery of molecular mechanisms underlying complex diseases.

Through extensive simulation studies, this study demonstrated that netRL outperforms existing methods in terms of network estimation and crucial edge selection. This study applied the proposed netRL framework to RNA-seq profiles obtained from Japan COVID-19 Task Force [13]. The current study aimed to uncover COVID-19 severity specific molecular interplays, especially between asymptomatic and critical samples. The results of this dataset successfully identified distinct gene regulatory networks for asymptomatic and critical COVID-19 groups, despite the significant sample size imbalance between these groups. In asymptomatic COVID-19 samples, the inferred networks were relatively dense, with strong connectivity among ribosomal proteins and translation-related genes. Importantly, ribosomal proteins were identified as phenotype-specific regulators in asymptomatic cases. Genes such as NFKBIA, B2M, CXCL8, and FOS emerged as common markers of critical and asymptomatic cases. Pathway enrichment analysis of the phenotype-specific networks provided additional biological insights. The network of COVID-19 critical samples was significantly enriched for “Positive regulation”-related GO terms, i.e., (“Positive regulation of macromolecule biosynthetic process,” “Positive regulation of cellular biosynthetic process” and “Positive regulation of biosynthetic process”). By contrast, the asymptomatic network was enriched for “Endoplasmic”-related (i.e., “Endoplasmic reticulum,” “Endoplasmic reticulum lumen”) and “Receptor complex” GO terms. By integrating prior biological knowledge into network inference, netRL was able to uncover robust and interpretable molecular signatures of COVID-19 progression that may have been overlooked by conventional differential expression approaches. These findings reinforce the value of the developed netRL in dissecting the molecular complexity of infectious disease.

The remainder of this paper is organized as follows. In the Methods section, this study introduced the formulation of netRL and describe its algorithmic implementation. Simulation studies are presented to compare the performance of netRL with existing approaches under various data scenarios. This study then applied the developed framework to COVID-19 transcriptomic data to demonstrate its practical utility in differential network analysis. Finally, this study discusses the biological implications of the findings and conclude with future perspectives.

## Methods

All data analyses and method development were performed during the period from May 2025 to December 2025. Different sample size scenarios can lead to various statistical challenges. In gene network inference and differential gene network analysis, unequal sample sizes may lead to bias and substantially reduce statistical power, particularly when applying methods such as Gaussian graphical models. Accordingly, the phenotype-specific molecular interactions observed under the condition of unequal sample sizes are more likely artifacts resulting from sampling imbalance rather than authentic biological differences. To address this issue, the current study propose a new computational method, network-constrained Random Lasso (netRL) that combines bootstrap resampling with random forest methodology in line with the random lasso framework [2]. By employing the random lasso framework, the proposed strategy can circumvent the challenges posed by unequal sample sizes in differential gene network analysis. Moreover, the developed framework integrates network biological knowledge into gene network inference, thereby enhancing the biological reliability of gene network analysis.

### Existing method for gene network estimation

The gene network can be formalized as a weighted graph *G* = (*V*,*E*,*W*), where *V* is the set of vertices corresponding to the *p* genes, E⊆V×V represents the set of edges describing gene-gene interactions, and *W* = (*w*_*ij*_) specifies the weights associated with each interaction (i,j)∈E. The normalized Laplacian matrix ***L*** for the graph *G* is given as [6,7],


$$\begin{array}{c} L=l_{ij}=\left\{ \begin{array}{cc} 1-\frac{w_{ij}}{d_{i}} & \text{if }i=j\text{ and }d_{i}\neq 0,\\ -\frac{w_{ij}}{\sqrt{d_{i}d_{j}}} & \text{if }(i,j)\in E,\\ 0 & \text{otherwise}, \end{array}\right. \end{array}$$ (1)

where $d_{i}=\sum_{i\sim j}^{w_{ij}}$ is the degree of each gene.

Suppose that $X=(x_{1},...,x_{n}{)}^{T}\in {\mathbb{R}}^{n\times p}$ is the n×p data matrix describing the expression levels of *p* genes. In this study, we considered a directed gene regulatory network that is often represented by the following linear regression model:


$$x_{i\ell }=\sum_{j\neq \ell }^{p}x_{ij}\beta_{j\ell }+\epsilon_{i\ell },\;\ell =1,...,p.$$

where $x_{i\ell }$ is the expression level of the $\ell^{th}$ gene in the *i*^th^ cell and $\epsilon_{i\ell }$ is the random error term accounting residual variation. Without loss of generality, this study assumed that the response has been centered and each predictor has been standardized,


$$\sum_{i=1}^{n}x_{i\ell }=0,\;\sum_{i=1}^{n}x_{ij}=0\;\text{and}\;\sum_{i=1}^{n}x_{ij}^{2}=0\;\text{for}\;j\neq \ell .$$

In order to estimate the gene regulatory network described by the linear regression model, the following *L*_1_-type regularization methods have often been used [1]:


$${\hat{\beta }}_{\ell }=\underset{\beta_{\ell }}{arg\;min}\{\frac{1}{2}\sum_{i=1}^{n}(x_{i\ell }-\sum_{j\neq \ell }^{p}x_{ij}\beta_{\ell j}{)}^{2}+\lambda P(|\beta_{\ell }|)\},$$ (2)

where λ>0 is a hyperparameter for controlling the degree of shrinkage of $\beta_{\ell }$ and $P(|\beta_{\ell }|)$ is the *L*_1_-type penalty, such as

- Lasso
  $$P(|\beta_{\ell }|)=\sum_{j\neq \ell }^{p}|\beta_{\ell j}|,$$
- Adaptive lasso
  $$P(|\beta_{\ell }|)=\sum_{j\neq \ell }^{p}w_{\ell j}|\beta_{\ell j}|,$$
  where $w_{\ell j}=1/|{\hat{\beta }}_{\ell j}^{OLS}|$ or $w_{\ell j}=1/|{\hat{\beta }}_{\ell j}^{ridge}|$ [1],
- Elastic net
  $$P(|\beta_{\ell }|)=\sum_{j\neq \ell }^{p}\{\frac{1}{2}(1-\delta )\beta_{\ell j}^{2}+\delta |\beta_{\ell j}|\}.$$
  where 0≤δ≤1 denotes the mixing parameter that balances the *L*_2_-norm penalty (i.e., ridge [8] and the *L*_1_-norm penalty (i.e., lasso).

By imposing *L*_1_-type penalties to the least squares loss, *L*_1_-type regularization methods simultaneously achieve edge selection and weight estimation.

However, the existing methods suffer from the following critical limitations in gene network inference.

- These methods suffer from unequal sample size issues, especially in uncovering phenotype specific characteristics, because, when *p* > *n*, the lasso and adaptive lasso can select at most *n* regulator genes [2]. When the sample size of a phenotype is relatively large, the corresponding model incorporates more substantial features, resulting in networks with an increased number of genes and their connections relative to those derived from limited samples. Hence, the identified phenotype-specific molecular interplays under the unequal sample sizes are more likely to reflect sampling imbalance rather than true biological differences.
- Furthermore, when constructing gene regulatory networks, many genes that are tightly connected within a biological pathway or functional module tend to exhibit strong correlation patterns. However, lasso tends to select only a few representatives while discarding the rest, thereby missing biologically meaningful groups.
- Although the elastic net alleviates some limitations of the lasso and adaptive lasso, it may produce biased network estimation results when handling highly correlated genes with regression coefficients of different magnitudes or opposing signs, owing to its grouping effect. Such situations frequently arise in gene network analysis, as genes within the same biological pathway are often strongly correlated but may contribute to the regulatory process with varying effect sizes or even opposite directions.

In summary, the existing approaches are not proper for gene network analysis, and gene network inference and differential gene network analysis between phenotypes should be conducted using methods that maintain robustness under consistent sample size conditions.

To overcome the critical limitations, this study considered the random lasso framework [2], which is one of *L*_1_-type regularization methods based on the bootstrapping samples and random forest technique. The random lasso exhibits superior capability in feature selection and model estimation relative to conventional *L*_1_-regularization approaches (such as lasso, adaptive lasso, and elastic net). Although the random lasso addresses several limitations of the existing approaches, it was originally developed from a purely statistical and computational perspective, without incorporating biological knowledge. This often poses challenges in interpreting the results of gene network inference.

### Network-constrained Random Lasso

This study developed a new computational strategy, called network-constrained Random Lasso (netRL), which explicitly incorporated network biological knowledge into the network inference procedure. In addition, this study proposed a dedicated edge selection scheme, implemented subsequent to the bootstrap resampling and random forest steps.

**Fig 1:**
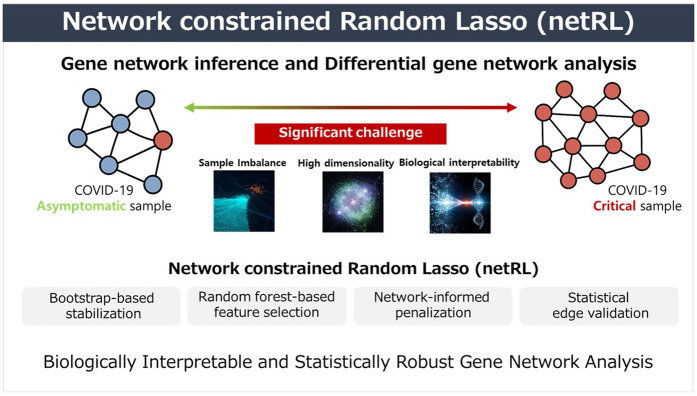
Overview of the network constrained Random Lasso (netRL).

The proposed netRL infers gene regulatory networks based on three stages: first, assessing the importance of regulator genes, second, gene network inference (edge selection and edge weight estimation), and third, assessing the significance of edges. Fig 1 shows the overview of the developed network constrained Random Lasso (netRL).

1. Generating gene importance measure
  (a) Suppose that there are two phenotypes (e.g., COVID-19 severe and non-severe) of samples, and wed estimate the gene network of the $\ell^{th}$ (ℓ=1,...p) target gene for the severe (or non-severe) samples. Draw Ω bootstrap samples with size $n=\text{min}(n_{S},n_{N})$ from the original dataset, where *n*_*S*_ and *n*_*N*_ are the sample sizes of the severe and non-severe groups, respectively.
  (b) For the $\omega_{1}^{th}$ bootstrap sample $\omega_{1}\in \{1,...,\Omega \}$, randomly select $p_{1}^{*}$ regulator genes, and apply the lasso to estimate the edge weight ${\hat{\beta }}_{\ell j}^{\left(\omega_{1}\right)}$ for $j=1,...,p_{1}^{*}$ for the $\ell^{th}$ target gene.
  (c) Compute the gene importance measure by incorporating the network biological knowledge. This study considered the widely used normalized centrality (i.e., hubenss: *H*_*j*_) and between centrality (*B*_*j*_) as indicators of gene’s importance [3,4].
    $$H_{j}=(\frac{1}{p_{1}^{*}-1}\sum_{k=1}^{p_{1}^{*}}a_{jk})\;\text{and}\;B_{j}=\frac{1}{\left(|V|-1)(|V|-2\right)}\sum_{t\neq j\neq u}\frac{b_{tu}(j)}{b_{tu}},$$ (3)
    where *a*_*jk*_ is the (*j*,*k*) element of the adjacency matrix, |*V*| is number of nodes (genes) in the network, *b*_*tu*_ is the total number of shortest paths from the *t*^th^ to *u*^th^ genes and *b*_*tu*_(*j*) is the number of these paths that pass through the *j*^th^ gene (where *j* is not an end point). The normalized centrality measures, i.e., hubness (*H*_*j*_) and betweenness centrality (*B*_*j*_), both take values in the interval [0,1], because hubness is defined as the sum of the *j*^th^ column of thhe adjacency matrix normalized by its maximum possible value $p^{*}-1$, and the betweenness centrality is scaled by the maximal attainable betweenness in a directed graph [5]. Hub genes are highly interconnected nodes that can propagate small local perturbations into global changes across the gene regulatory network, underscoring their functional importance. Accordingly, hubness serves as an indicator of a gene’s essentiality. In parallel, “betweenness centrality” evaluates a gene’s importance by measuring its control over information flow between other genes in the network. The proposed strategy incorporated the centralities to evaluate the importance of a gene and propose the following gene importance measure
    $$C_{j}=(\frac{H_{j}+B_{j}}{2})|\frac{1}{\Omega }\sum_{\omega_{1}=1}^{\Omega }{\hat{\beta }}_{\ell j}^{\left(\omega_{1}\right)}|.$$ (4)
    The gene importance measure can be considered as the weighted version of absolute value of average bootstrap coefficient (i.e., $\left|\frac{1}{\Omega }\sum_{\omega_{1}=1}^{\Omega }{\hat{\beta }}_{\ell j}^{\left(\omega_{1}\right)}\right|$) that indicates crucialness of genes in the statistical viewpoint. The weighting factor constructed from standardized centrality metrics takes values in [0,1], ensuring that it modulates the bootstrap coefficient without altering its scale. That is, the proposed strategy measures the importance of genes based on not only statistical but also network biology perspectives, and thus, the proposed method can perform biologically reliable gene network inference.
2. Gene network estimation
  (a) Compute the normalized Laplacian matrix ***L*** based on the following weighted adjacency matrix
    $$W=w_{kj}=w_{jk}=\frac{\left|{\hat{\beta }}_{kj}^{\left(*\right)}|+|{\hat{\beta }}_{jk}^{\left(*\right)}\right|}{2},$$ (5)
    where ${\hat{\beta }}_{kj}^{\left(*\right)}=\frac{1}{\Omega }\sum_{\omega_{1}}^{\Omega }{\hat{\beta }}_{kj}^{\left(\omega_{1}\right)}$. The weighted adjacency matrix can be also defined by an external prior network, e.g., gene–gene correlation matrix, where each entry *w*_*kj*_ corresponds to the correlation coefficient between the expression levels of *k*^th^ and *j*^th^ genes.
  (b) Draw another set of Ω bootstrap samples with size $n=\text{min}(n_{S},n_{N})$ by sampling from the original dataset.
  (c) For the $\omega_{2}^{th}$ bootstrap sample $\omega_{2}\in \{1,...,\Omega \}$, randomly select $p_{2}^{*}$ candidate regulator genes with selection probability of the *j*^th^ regulator gene proportional to its importance *C*_*j*_.
  (d) Construct the normalized Laplacian matrix for the randomly selected $p_{2}^{*}$ genes from the computed ***L*** in stage 2.a, i.e., $L^{\left(\omega_{2}\right)}\in {\mathbb{R}}^{p_{2}^{*}\times p_{2}^{*}}$.
  (e) For the interpretable and biologically reliable gene network estimation, this study considered the following network constrained *L*_1_-type regularization method [6],
    $${\hat{\beta }}_{\ell }=\underset{\beta_{\ell }}{arg\;min}\{\frac{1}{2}\sum_{i=1}^{n}(x_{i\ell }-\sum_{j\neq \ell }^{p_{2}^{*}}x_{ij}\beta_{\ell j}{)}^{2}+\lambda_{1}\sum_{j\neq \ell }^{p_{2}^{*}}|\beta_{\ell }|+\lambda_{2}\beta_{\ell }L^{\left(\omega_{2}\right)}\beta_{\ell }\}$$ (6)
    $$\begin{array}{cc} & =\underset{\beta_{\ell }}{arg\;min}\{\frac{1}{2}\sum_{i=1}^{n}(x_{i\ell }-\sum_{j\neq \ell }^{p_{2}^{*}}x_{ij}\beta_{\ell j}{)}^{2}\\ & +\lambda_{1}\sum_{j\neq \ell }^{p_{2}^{*}}|\beta_{\ell }|+\lambda_{2}\sum_{k=1}^{p_{2}^{*}}\sum_{j=1}^{p_{2}^{*}}w_{kj}(\frac{\text{sgn}(\beta_{\ell k})\beta_{\ell k}}{\sqrt{d_{k}}}-\frac{\text{sgn}(\beta_{\ell j})\beta_{\ell j}}{\sqrt{d_{j}}}{)}^{2}\}. \end{array}$$ (7)
    As genes within the same network neighborhood tend to share functional roles, this study applied *w*_*kj*_ in the second penalty term to encourage consistency among the estimated coefficients. This penalty induces local smoothing across the network, thereby promoting the simultaneous selection of biologically related genes. Moreover, scaling gene coefficients by the square root of node degree enables the method to impose relatively weaker penalties on highly connected hub genes. Consequently, hub genes together with their neighboring nodes were more likely to receive larger coefficients, thereby increasing their chance of being selected during network reconstruction [6]. The proposed strategy applied the network constrained *L*_1_-type regularization method to the $\omega_{2}^{th}$ bootstrap samples for the selected $p_{2}^{*}$ genes and estimate ${\hat{\beta }}_{\ell j}^{\left(\omega_{2}\right)}$, $j=1,...,p_{2}^{*}$.
  (f) Compute edge weights of *p* regulator genes for the $\ell^{th}$ target gene
    $${\hat{\beta }}_{\ell j}=\frac{1}{\Omega }\sum_{\omega_{2}=1}^{\Omega }{\hat{\beta }}_{\ell j}^{\left(\omega_{2}\right)},\;j=1,...,p.$$ (8)
3. Access significance of edges
  The procedures of stages 1 and 2 have the potential to generate false-positive regulator gene selections, owing to the fact that the final edge weight estimation in (8) is derived from the averaged coefficients over Ω iterations. Hence, regulator genes exhibit nonzero coefficients only in a model among the Ω models, resulting in nonzero edge weights. Thus, the assessment of significance should be performed following the bootstrap-based edge estimation procedure.
  (a) Permutation test
    To enhance the effectiveness of edge selection, this study proposed a novel strategy that evaluates the significance of edges using the permutation test. As a first step, this study quantifies the selection frequency of the *j*^th^ regulator gene is selected among the Ω bootstrap models as follows,
    $$m_{\ell j}=\sum_{\omega_{2}=1}^{\Omega }I({\hat{\beta }}_{\ell j}^{\left(\omega_{2}\right)}\neq 0),$$
    where I(·) is the indicator function. To compute the selection frequency of the *j*^th^ regulator gene under the permutation framework, the proposed strategy permutes the expression levels of target genes and re-estimates the gene networks following “Step 2: Gene network estimation,” yielding ${\hat{\beta }}_{\ell j}^{\left(\omega_{2}\right)}(\phi )$ for ϕ=1,...,Π for Π permutation replicates. Then, the permutation selection frequency is computed as follows,
    $$m_{\ell j}(\phi )=\sum_{\omega_{2}=1}^{\Omega }I({\hat{\beta }}_{\ell j}^{\left(\omega_{2}\right)}(\phi )\neq 0),\;\phi =1,...,\Pi .$$
    Next, the proposed strategy computed the permutation p-value as follows:
    $${\text{p.value}}_{\ell j}=\frac{\sum_{\phi =1}^{\Pi }I(m_{\ell j}(\phi )\geq m_{\ell j})}{\Pi }.$$ (9)
    The proposed netRL selected regulator genes with p-values less than or equal to a significance level α.
  (b) Using percentile bootstrap interval
    This study also compared the netRL based on significance assessment of edges based on percentile bootstrap interval [11]. The confidence interval was determined as follows,
    $$({\hat{\beta }}_{\ell j}(\frac{\alpha }{2}),{\hat{\beta }}_{\ell j}(1-\frac{\alpha }{2})).$$ (10)
    where ${\hat{\beta }}_{\ell j}(\frac{\alpha }{2})$ and ${\hat{\beta }}_{\ell j}(1-\frac{\alpha }{2})$ are $\frac{\alpha }{2}$ and $1-\frac{\alpha }{2}$ quantiles of the generated bootstrap replications, respectively. Under a significance threshold of α, genes whose (1−α)% confidence intervals for $\beta_{\ell j}$ encompass zero were discarded from the list of candidate regulators of the $\ell^{th}$ target gene.

The proposed netRL framework introduces several methodological advances over existing approaches, which can be summarized as follows.

- *Integrating random lasso with Laplacian-regularized regression to handle sample imbalance*: The incorporation of a random lasso framework into Laplacian-regularized regression is a key design choice of netRL. This integration enables us to mitigate severe sample imbalance across phenotypes while improving the stability of regulator gene selection. Although numerous statistical methodologies have been developed and used to gene network estimation (e.g., sparse Gaussian Graphical Models (GGMs) [9], Laplacian-regularized regressions [6], etc.), existing approaches typically assume comparable sample sizes across conditions. Under unequal-sample scenarios, these methods tend to identify spurious molecular interactions driven by sampling imbalance rather than true biological differences. By repeatedly fitting Laplacian-regularized models on balanced bootstrap subsamples, netRL explicitly addresses this issue, yielding more robust phenotype-specific network inference.
- *Centrality-weighted subsampling to quantify gene importance within the random lasso framework*: Traditional stability selection [10] and random lasso [2] approaches rely on uniform subsampling of predictors and do not incorporate biological or network-level information. Moreover, although prior Laplacian-regularized regression and sparse GGM methods exploit network structure as a constraint during the estimation stage, they do not use network centrality information to guide the predictor subsampling process. In contrast, netRL directly integrates biological network knowledge into the subsampling mechanism by quantifying gene-level importance through a combination of bootstrap-based coefficient stability and graph-derived structural centrality measures, such as hubness and betweenness. This approach yields a biologically meaningful sampling distribution that preferentially selects genes occupying topologically influential positions within the underlying network.
- *Two-stage inference and selection with hypergeometric calibration*: Rather than relying solely on coefficient magnitudes or regularization paths, the proposed netRL employs a two-stage inference scheme. In the second stage, a hypergeometric test is used to assess whether edges repeatedly selected across bootstrap replicates occur more frequently than expected by chance under random subsampling. This calibration step addresses a known limitation of random lasso–based feature selection, namely the lack of a formal statistical criterion for edge significance, and provides principled control over false-positive discoveries.

### Monte Carlo simulation

Monte Carlo simulation experiments were performed with simulated datasets to investigate the performance of the proposed netRL. In line with earlier studies on network-constrained regularization method [6], this study adopted benchmark settings to establish simulation scenarios.

This study supposed *u* transcription factors (TFs) and each regulates *v* genes, and the expression levels of TFs follows standard normal distribution, i.e., $x_{i,TF_{j}}\sim N(0,1),j=1,...,u$. The expression levels of the TF and the regulated gene by the TF are jointly distributed as a bivariate normal with a correlation of 0.7, i.e., the regulated gene by the *j*^th^ TF follows $x_{i,rTF_{j}}\sim N(0.7\times x_{i,TFj},0.51)$. This study defined the expression data for the u×(v+1) genes as follows,


$$x_{i}=(x_{i,TF_{1}},\underset{v}{\underbrace{x_{i,rTF_{11}},\cdots ,x_{i,rTF_{1v}}}},\cdots ,x_{i,TF_{u}},\underset{v}{\underbrace{x_{i,rTF_{u1}},\cdots ,x_{i,rTF_{uv}}}}{)}^{T}.$$ (11)

Here, this study assumed that the u×(v+1) genes as regulator genes and they regulate the $\ell^{th}$ target gene as follows,


$$y_{i\ell }=\sum_{j=1}^{u\times (v+1)}x_{ij}\beta_{\ell j}+\epsilon_{i\ell },$$ (12)

where $\epsilon_{i\ell }\sim N(0,\sigma^{2})$.

The current study explored multiple possible configurations of the gene regulatory structure for the $\ell^{th}$ target gene as described below.

- Scenario 1:

$$\beta_{\ell }=(5,\underset{v}{\underbrace{\frac{5}{\sqrt{10}},...,\frac{5}{\sqrt{10}}}},-5,\underset{v}{\underbrace{\frac{-5}{\sqrt{10}},...,\frac{-5}{\sqrt{10}}}},3,\underset{v}{\underbrace{\frac{3}{\sqrt{10}},...,\frac{3}{\sqrt{10}}}},-3,\underset{v}{\underbrace{\frac{-3}{\sqrt{10}},...,\frac{-3}{\sqrt{10}}}},0,...,0),$$

- Scenario 2:

$$\begin{array}{cc} \beta_{\ell }=( & 5,\underset{v\times 0.3}{\underbrace{\frac{-5}{\sqrt{10}},...,\frac{-5}{\sqrt{10}}}},\underset{v\times 0.7}{\underbrace{\frac{5}{\sqrt{10}},...,\frac{5}{\sqrt{10}}}},-5,\underset{v\times 0.3}{\underbrace{\frac{5}{\sqrt{10}},...,\frac{5}{\sqrt{10}}}},\underset{v\times 0.7}{\underbrace{\frac{-5}{\sqrt{10}},...,\frac{-5}{\sqrt{10}}}},\\ & 3,\underset{v\times 0.3}{\underbrace{\frac{-3}{\sqrt{10}},...,\frac{-3}{\sqrt{10}}}}\underset{v\times 0.7}{\underbrace{\frac{3}{\sqrt{10}},...,\frac{3}{\sqrt{10}}}},-3,\underset{v\times 0.3}{\underbrace{\frac{3}{\sqrt{10}},...,\frac{3}{\sqrt{10}}}},\underset{v\times 0.7}{\underbrace{\frac{-3}{\sqrt{10}},...,\frac{-3}{\sqrt{10}}}},0,...,0), \end{array}$$

- Scenario 3:

$$\beta_{\ell }=(5,\underset{v}{\underbrace{\frac{5}{\sqrt{5}},...,\frac{5}{\sqrt{5}}}},-5,\underset{v}{\underbrace{\frac{-5}{\sqrt{15}},...,\frac{-5}{\sqrt{15}}}},3,\underset{v}{\underbrace{\frac{3}{\sqrt{5}},...,\frac{3}{\sqrt{5}}}},-3,\underset{v}{\underbrace{\frac{-3}{\sqrt{15}},...,\frac{-3}{\sqrt{15}}}},0,...,0)$$

- Scenario 4:

$$\begin{array}{cc} \beta_{\ell }=( & 5,\underset{v\times 0.3}{\underbrace{\frac{-5}{\sqrt{5}},...,\frac{-5}{\sqrt{5}}}},\underset{v\times 0.7}{\underbrace{\frac{5}{\sqrt{5}},...,\frac{5}{\sqrt{5}}}},-5,\underset{v\times 0.3}{\underbrace{\frac{5}{\sqrt{15}},...,\frac{5}{\sqrt{15}}}},\underset{v\times 0.7}{\underbrace{\frac{-5}{\sqrt{15}},...,\frac{-5}{\sqrt{15}}}},\\ & 3,\underset{v\times 0.3}{\underbrace{\frac{-3}{\sqrt{5}},...,\frac{-3}{\sqrt{5}}}}\underset{v\times 0.7}{\underbrace{\frac{3}{\sqrt{5}},...,\frac{3}{\sqrt{5}}}},-3,\underset{v\times 0.3}{\underbrace{\frac{3}{\sqrt{15}},...,\frac{3}{\sqrt{15}}}},\underset{v\times 0.7}{\underbrace{\frac{-3}{\sqrt{15}},...,\frac{-3}{\sqrt{15}}}},0,...,0). \end{array}$$

This study considered σ=1,3, number of TFs *u* = 10, 20, and simulated 50 datasets consisting of *n* = 60 observations from the 4 scenarios, where the training and test datasets consisted of 80% (48) and 20% (12) observations, respectively. This study also considered two situations in which the number of crucial regulator genes corresponding $\beta_{\ell j}\neq 0$ is smaller (i.e., *u* = 10: Situation 1) and larger (i.e., *u* = 20: Situation 2) than the number of observations of the training dataset (i.e., *n* = 48).

**Fig 2:**
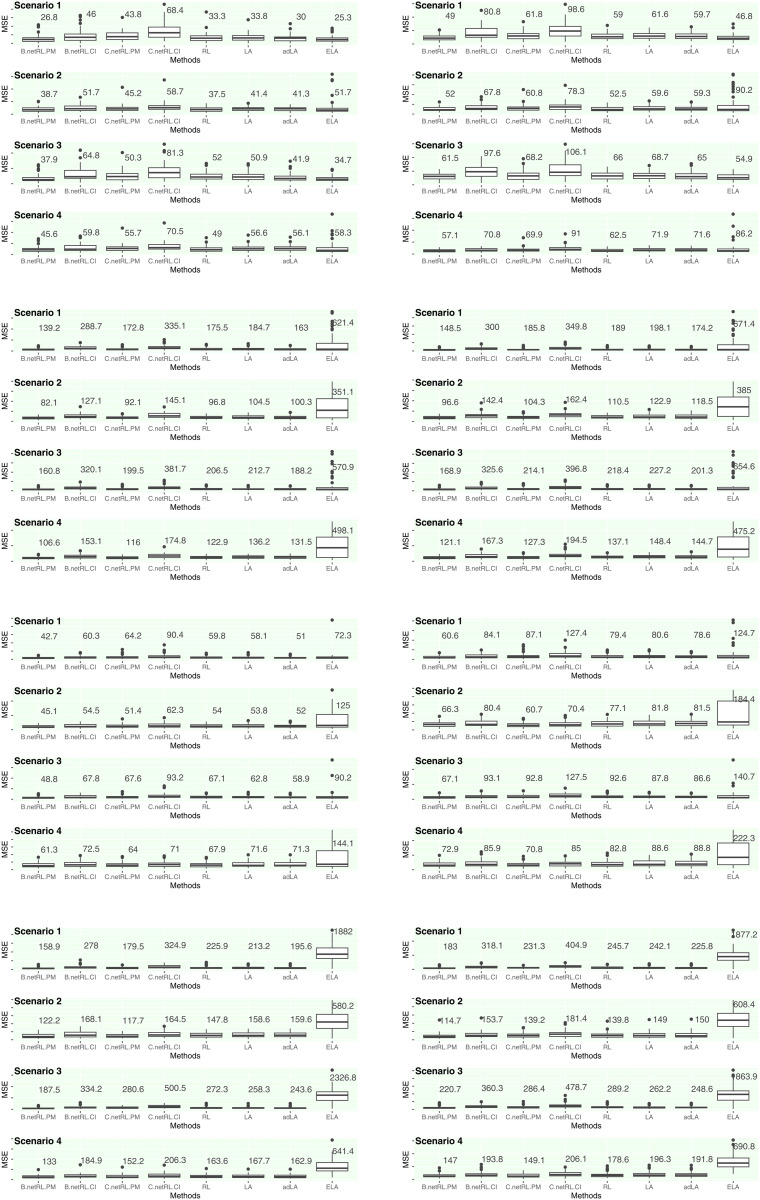
Mean square error for estimating expression levels of the target gene in test dataset (netRL.PM: netRL with permutation test and netRL. CI: netRL with bootstrap confidence interval, where B.netRL and C.netRL indicate netRL based on constructed Laplacian matrix by regression coefficient and correlation coefficient, respectively. RL: ordinary random lasso, LA: lasso, adLA: adaptive lasso, ELA: elastic net).

To evaluate the effectiveness of netRL, this study compared its performance against several established *L*_1_-type regularization methods, namely lasso, adaptive lasso, elastic net, and standard random lasso, where netRL with boostrap interval is based on 95% confidence interval. The hyperparameters (i.e., $\lambda_{1}$ and λ2) of the *L*_1_-type regularization methods were selected by using the following Bayesian Information Criterion (BIC) [12],


$$\text{BIC}=\frac{||x_{\ell }-{\hat{x}}_{\ell }|{|}^{2}}{n\sigma^{2}}+\frac{\text{\{log}(n)}{\}}n\hat{df},$$ (13)

where $x_{\ell }=(x_{1\ell },...,x_{n\ell }{)}^{T}$ is expression levels of the $\ell^{th}$ gene considered as a target gene, $\hat{df}$ is the degree of freedom of the estimated model of the gene network of the $\ell^{th}$ gene. For the tuning parameters $\lambda_{1}$ and $\lambda_{2}$, their optimal values are determined via a grid search that selects the combination minimizing the BIC. This study estimated gene networks based on *B* = 200 bootstrap replications. Fig 2 shows the mean square error (MSE) in estimating expression levels of target genes based on the test dataset. As illustrated in Fig 2, the proposed netRL demonstrated superior performance in gene network estimation, with netRL combined with the hypergeometric test achieving the most effective results. However, incorporating the bootstrap confidence interval into netRL does not yield satisfactory results. In scenario 2, the elastic net demonstrated notably weak results. This study also evaluated the edge selection results, i.e., regulator gene selection in the model of the $\ell^{th}$ target gene. Tables 1 and 2 present the true positive rate (TPR), true negative rate (TNR), and overall accuracy for regulatory gene selection, with the most effective results highlighted in bold, for situations 1 and 2, respectively. Consistent with the MSE results, the integration of netRL with the hypergeometric test and median resulted in effective crucial gene selection. The developed strategy combining the hypergeometric test and median yielded similar outcomes in Situation 1; however, the hypergeometric test–based method demonstrated clear advantages in Situation 2. Although netRL with bootstrap confidence intervals achieved favorable performance in terms of true positive rate, it failed to effectively filter out noise regulator genes with zero coefficients ($\beta_{\ell j}=0$), especially in Situation 2. The results indicate that netRL has strong potential as an effective approach for biologically meaningful gene network inference. This study also considered a more conservative strategy based on an inner bootstrap (nested resampling) scheme, in which the Laplacian matrix is constructed within each inner $\omega_{2}^{th}$ bootstrap replication using randomly selected samples and variables, and subsequently applied in the outer regression step. Although this strategy was also evaluated, its results are not described in the main text because the method did not perform well in practice. For completeness, the corresponding results are provided in the Supplementary Materials.

**Table 1:** Simulation results of Situation 1: Regulator selection accuracy.

	σ	♯ TFs	SN	netRL: Beta		netRL: Corr		RL	LASSO	adLASSO	ELA
				PM	CI	PM	CI				
TPR	1	10	1	0.87	0.65	0.91	0.69	0.86	0.65	0.65	0.95
			2	0.77	0.45	0.72	0.45	0.77	0.54	0.54	0.71
			3	0.95	0.67	0.90	0.66	0.86	0.65	0.65	0.93
			4	0.74	0.44	0.70	0.43	0.78	0.53	0.53	0.66
		20	1	0.93	0.70	0.89	0.64	0.81	0.59	0.59	0.91
			2	0.73	0.49	0.72	0.47	0.68	0.46	0.46	0.38
			3	0.92	0.69	0.89	0.62	0.81	0.58	0.58	0.88
			4	0.73	0.47	0.69	0.45	0.69	0.47	0.47	0.37
	3	10	1	0.93	0.62	0.87	0.60	0.86	0.63	0.63	0.91
			2	0.76	0.41	0.70	0.41	0.78	0.52	0.52	0.60
			3	0.91	0.60	0.86	0.61	0.86	0.63	0.63	0.90
			4	0.72	0.41	0.69	0.39	0.78	0.51	0.51	0.57
		20	1	0.91	0.66	0.87	0.59	0.80	0.56	0.56	0.80
			2	0.72	0.46	0.70	0.43	0.68	0.43	0.43	0.21
			3	0.91	0.66	0.86	0.61	0.80	0.56	0.56	0.80
			4	0.71	0.45	0.69	0.39	0.68	0.44	0.44	0.17
TNR	1	10	1	0.76	0.89	0.88	0.99	0.79	0.92	0.92	0.87
			2	0.79	0.99	0.81	0.98	0.58	0.81	0.81	0.72
			3	0.84	0.99	0.89	0.99	0.78	0.93	0.93	0.87
			4	0.78	0.99	0.81	0.98	0.56	0.82	0.82	0.73
		20	1	0.89	0.99	0.88	0.99	0.85	0.94	0.94	0.87
			2	0.83	0.98	0.82	0.98	0.76	0.90	0.90	0.90
			3	0.89	0.99	0.89	0.99	0.84	0.94	0.94	0.88
			4	0.82	0.98	0.82	0.98	0.74	0.90	0.90	0.90
	3	10	1	0.82	0.99	0.87	0.99	0.71	0.89	0.89	0.81
			2	0.76	0.99	0.78	0.98	0.52	0.80	0.80	0.73
			3	0.82	0.99	0.87	0.99	0.71	0.90	0.90	0.82
			4	0.75	0.98	0.78	0.98	0.51	0.79	0.79	0.74
		20	1	0.87	0.99	0.86	0.99	0.81	0.93	0.93	0.87
			2	0.81	0.98	0.81	0.98	0.72	0.89	0.89	0.95
			3	0.87	0.99	0.87	0.99	0.80	0.93	0.93	0.87
			4	0.80	0.98	0.79	0.98	0.71	0.89	0.89	0.95
FDR	1	10	1	0.15	0.28	0.09	0.24	0.24	0.33	0.33	0.16
			2	0.13	0.23	0.08	0.20	0.27	0.32	0.32	0.14
			3	0.06	0.25	0.10	0.25	0.16	0.28	0.28	0.06
			4	0.25	0.36	0.27	0.37	0.30	0.37	0.37	0.31
		20	1	0.07	0.23	0.11	0.27	0.20	0.31	0.31	0.12
			2	0.06	0.20	0.10	0.24	0.20	0.30	0.30	0.08
			3	0.08	0.24	0.11	0.28	0.19	0.31	0.31	0.14
			4	0.25	0.35	0.27	0.36	0.30	0.38	0.38	0.38
	3	10	1	0.08	0.28	0.13	0.29	0.18	0.30	0.30	0.10
			2	0.07	0.24	0.12	0.25	0.21	0.30	0.30	0.08
			3	0.10	0.29	0.14	0.28	0.18	0.30	0.30	0.12
			4	0.27	0.38	0.28	0.38	0.32	0.38	0.38	0.35
		20	1	0.10	0.25	0.13	0.29	0.19	0.33	0.33	0.19
			2	0.08	0.22	0.11	0.26	0.18	0.31	0.31	0.14
			3	0.10	0.26	0.13	0.28	0.19	0.32	0.32	0.18
			4	0.26	0.36	0.28	0.38	0.31	0.39	0.39	0.44
ACC	1	10	1	0.81	0.77	0.90	0.84	0.82	0.79	0.79	0.91
			2	0.78	0.72	0.77	0.72	0.67	0.68	0.68	0.71
			3	0.90	0.83	0.90	0.83	0.82	0.79	0.79	0.90
			4	0.76	0.71	0.75	0.71	0.67	0.67	0.67	0.70
		20	1	0.91	0.85	0.88	0.82	0.83	0.76	0.76	0.89
			2	0.78	0.73	0.77	0.72	0.72	0.68	0.68	0.64
			3	0.90	0.84	0.89	0.81	0.82	0.76	0.76	0.88
			4	0.78	0.72	0.76	0.71	0.71	0.68	0.68	0.64
	3	10	1	0.88	0.81	0.87	0.79	0.78	0.76	0.76	0.86
			2	0.76	0.70	0.74	0.69	0.65	0.66	0.66	0.67
			3	0.86	0.79	0.87	0.80	0.79	0.76	0.76	0.86
			4	0.74	0.70	0.74	0.68	0.64	0.65	0.65	0.66
		20	1	0.89	0.82	0.87	0.79	0.80	0.74	0.74	0.83
			2	0.77	0.72	0.75	0.70	0.70	0.66	0.66	0.58
			3	0.89	0.82	0.87	0.80	0.80	0.74	0.74	0.84
			4	0.76	0.71	0.74	0.68	0.70	0.66	0.66	0.56
♯ Edges	1	10	1	54	36	48	31	51	34	34	50
			2	47	20	44	21	62	36	36	50
			3	52	30	47	30	53	34	34	49
			4	47	20	43	20	64	35	35	47
		20	1	61	33	61	30	63	36	36	62
			2	62	25	64	24	72	38	38	34
			3	60	32	59	30	64	35	35	59
			4	63	24	62	23	76	38	38	33
	3	10	1	53	28	47	27	57	35	35	53
			2	49	19	46	19	66	36	36	44
			3	52	27	47	28	57	35	35	52
			4	48	19	45	18	67	36	36	42
		20	1	63	32	63	29	69	37	37	59
			2	66	24	64	23	79	38	38	18
			3	62	32	61	29	70	37	37	58
			4	66	24	66	22	81	39	39	16

**Table 2:** Simulation results of Situation 2: Regulator selection accuracy.

	σ	♯ TFs	SN	netRL: Beta		netRL: Corr		RL	LASSO	adLASSO	ELA
				PM	CI	PM	CI				
TPR	1	10	1	0.82	0.47	0.79	0.45	0.70	0.39	0.39	0.47
			2	0.64	0.34	0.62	0.31	0.64	0.35	0.35	0.22
			3	0.80	0.47	0.78	0.45	0.71	0.38	0.38	0.53
			4	0.63	0.32	0.61	0.30	0.62	0.34	0.34	0.17
		20	1	0.87	0.53	0.83	0.46	0.64	0.34	0.34	0.05
			2	0.68	0.34	0.66	0.32	0.55	0.29	0.29	0.01
			3	0.86	0.52	0.81	0.45	0.66	0.35	0.35	0.21
			4	0.66	0.32	0.65	0.30	0.55	0.29	0.29	0.00
	3	10	1	0.80	0.45	0.78	0.44	0.69	0.38	0.38	0.48
			2	0.62	0.31	0.61	0.30	0.61	0.34	0.34	0.30
			3	0.80	0.46	0.77	0.44	0.70	0.37	0.37	0.52
			4	0.61	0.31	0.60	0.29	0.62	0.33	0.33	0.18
		20	1	0.84	0.49	0.80	0.42	0.65	0.35	0.35	0.08
			2	0.67	0.34	0.67	0.32	0.54	0.29	0.29	0.03
			3	0.82	0.48	0.78	0.43	0.65	0.34	0.34	0.09
			4	0.64	0.32	0.65	0.30	0.56	0.29	0.29	0.01
TNR	1	10	1	0.93	1.00	0.93	0.99	0.87	0.97	0.97	0.97
			2	0.87	0.99	0.87	0.99	0.79	0.93	0.93	0.95
			3	0.94	1.00	0.93	0.99	0.87	0.97	0.97	0.96
			4	0.86	0.99	0.87	0.99	0.76	0.92	0.92	0.96
		20	1	0.92	0.99	0.91	1.00	0.90	0.98	0.98	1.00
			2	0.87	0.99	0.86	0.99	0.86	0.96	0.96	1.00
			3	0.92	0.99	0.90	0.99	0.90	0.98	0.98	0.99
			4	0.86	0.99	0.85	0.99	0.85	0.96	0.96	1.00
	3	10	1	0.93	1.00	0.93	0.99	0.88	0.97	0.97	0.96
			2	0.85	0.99	0.86	0.99	0.78	0.92	0.92	0.93
			3	0.93	0.99	0.93	0.99	0.85	0.97	0.97	0.95
			4	0.85	0.99	0.86	0.98	0.75	0.92	0.92	0.95
		20	1	0.91	0.99	0.90	0.99	0.90	0.98	0.98	0.99
			2	0.86	0.99	0.86	0.99	0.86	0.95	0.95	1.00
			3	0.91	0.99	0.91	0.99	0.89	0.97	0.97	1.00
			4	0.86	0.99	0.85	0.99	0.85	0.96	0.96	1.00
FDR	1	10	1	0.16	0.35	0.19	0.36	0.26	0.39	0.39	0.31
			2	0.15	0.32	0.17	0.33	0.27	0.39	0.39	0.26
			3	0.17	0.35	0.19	0.36	0.26	0.39	0.39	0.29
			4	0.30	0.41	0.31	0.41	0.32	0.41	0.41	0.45
		20	1	0.13	0.32	0.15	0.35	0.27	0.40	0.40	0.48
			2	0.11	0.28	0.14	0.32	0.26	0.39	0.39	0.46
			3	0.14	0.33	0.17	0.36	0.27	0.40	0.40	0.47
			4	0.28	0.41	0.29	0.41	0.35	0.43	0.43	0.50
	3	10	1	0.18	0.35	0.19	0.36	0.26	0.39	0.39	0.32
			2	0.16	0.33	0.18	0.33	0.26	0.39	0.39	0.26
			3	0.18	0.35	0.20	0.36	0.26	0.39	0.39	0.30
			4	0.31	0.41	0.32	0.42	0.33	0.42	0.42	0.45
		20	1	0.15	0.34	0.18	0.37	0.29	0.41	0.41	0.48
			2	0.14	0.31	0.17	0.34	0.27	0.40	0.40	0.47
			3	0.16	0.34	0.20	0.37	0.28	0.40	0.40	0.47
			4	0.29	0.41	0.29	0.42	0.35	0.43	0.43	0.50
ACC	1	10	1	0.87	0.73	0.86	0.72	0.79	0.68	0.68	0.72
			2	0.75	0.66	0.74	0.65	0.71	0.64	0.64	0.59
			3	0.87	0.73	0.86	0.72	0.79	0.67	0.67	0.75
			4	0.74	0.66	0.74	0.64	0.69	0.63	0.63	0.56
		20	1	0.89	0.76	0.87	0.73	0.77	0.66	0.66	0.52
			2	0.77	0.66	0.76	0.65	0.71	0.62	0.62	0.51
			3	0.89	0.76	0.86	0.72	0.78	0.66	0.66	0.60
			4	0.76	0.65	0.75	0.64	0.70	0.62	0.62	0.50
	3	10	1	0.86	0.72	0.85	0.72	0.79	0.67	0.67	0.72
			2	0.74	0.65	0.73	0.64	0.70	0.63	0.63	0.62
			3	0.86	0.73	0.85	0.72	0.77	0.67	0.67	0.74
			4	0.73	0.65	0.73	0.64	0.68	0.63	0.63	0.57
		20	1	0.87	0.74	0.85	0.71	0.77	0.66	0.66	0.54
			2	0.77	0.66	0.76	0.65	0.70	0.62	0.62	0.52
			3	0.87	0.74	0.84	0.71	0.77	0.65	0.65	0.54
			4	0.75	0.65	0.75	0.64	0.70	0.62	0.62	0.50
♯ Edges	1	10	1	77	40	75	38	75	36	36	43
			2	71	30	69	28	81	38	38	24
			3	76	40	74	39	75	35	35	49
			4	70	29	68	27	83	39	39	19
		20	1	100	47	100	40	88	37	37	4
			2	101	33	104	31	93	39	39	1
			3	98	45	101	40	88	37	37	20
			4	102	31	103	30	96	39	39	0
	3	10	1	76	39	75	38	74	36	36	46
			2	71	28	69	27	79	39	39	34
			3	76	39	74	38	78	36	36	50
			4	70	28	69	26	84	38	38	22
		20	1	100	43	101	38	89	37	37	9
			2	103	32	104	31	93	39	39	4
			3	100	43	97	38	92	37	37	9
			4	101	31	106	29	99	39	39	1

This study next evaluated the computational efficiency of the proposed netRL in comparison with the existing methods. Table 3 shows the running times for the gene networks, i.e., estimation of the model in (11), where running time of the bootstrap-based strategies were evaluated based on 200 bootstrap replications. As shown in Table 3, the bootstrap-based strategies (e.g., random lasso and the proposed netRL) exhibit higher computational complexity compared with ordinary *L*_1_-type regularization methods (i.e., lasso, adaptive lasso, and elastic net). However, the increased computational cost remains acceptable given the improved stability and performance of the proposed approach.

**Table 3:** Running time (in seconds) of the gene network estimation by using various methods. The running time of the bootstrap-based strategies were evaluated based on 200 bootstrap replications.

	σ	♯ TFs	SN	netRL: Beta		netRL: Corr		RL	LASSO	adLASSO	ELA
				PM	CI	PM	CI				
Situation 1	1	10	1	87.68	87.59	128.53	128.36	87.57	0.00	0.17	0.02
			2	110.59	110.42	150.15	150.04	110.40	0.02	0.23	0.06
			3	116.42	116.29	146.33	146.21	116.28	0.00	0.20	0.05
			4	118.09	117.97	137.94	137.83	117.95	0.00	0.22	0.03
		20	1	306.83	306.47	383.53	383.28	306.44	0.00	0.83	0.03
			2	371.67	371.40	358.77	358.47	371.37	0.00	0.91	0.04
			3	350.97	350.77	344.98	344.73	350.72	0.02	0.94	0.04
			4	354.67	354.46	279.33	279.09	354.43	0.00	0.94	0.03
	3	10	1	113.83	113.66	145.58	145.42	113.61	0.00	0.25	0.03
			2	124.84	124.75	152.02	151.82	124.73	0.00	0.14	0.03
			3	117.48	117.39	141.95	141.84	117.38	0.02	0.19	0.03
			4	130.99	130.90	143.69	143.57	130.88	0.01	0.15	0.03
		20	1	304.27	304.02	380.31	380.07	303.95	0.01	0.75	0.03
			2	378.86	378.61	357.25	356.94	378.56	0.00	1.05	0.07
			3	348.33	348.10	343.86	343.66	348.06	0.02	0.82	0.05
			4	358.62	358.36	279.59	279.40	358.33	0.00	0.85	0.03
Situation 2	1	10	1	308.13	307.89	364.63	364.36	307.86	0.00	0.83	0.03
			2	381.19	380.83	340.35	340.06	380.78	0.01	1.31	0.05
			3	373.44	373.06	331.46	331.21	373.01	0.00	1.11	0.04
			4	372.86	372.59	274.00	273.78	372.53	0.00	1.09	0.06
		20	1	1030.68	1030.14	1002.59	1001.98	1030.06	0.02	5.98	0.07
			2	885.42	885.09	684.75	684.46	885.06	0.00	3.48	0.05
			3	615.86	615.51	570.84	570.50	615.47	0.00	3.61	0.03
			4	676.03	675.67	621.14	620.83	675.63	0.02	4.55	0.06
	3	10	1	312.56	312.32	358.82	358.58	312.29	0.00	1.00	0.03
			2	393.64	393.39	336.41	336.14	393.34	0.02	1.12	0.06
			3	373.67	373.32	329.55	329.31	373.28	0.00	1.08	0.04
			4	371.27	371.04	273.80	273.58	370.97	0.00	0.87	0.04
		20	1	1032.27	1031.71	954.25	953.73	1031.61	0.01	5.83	0.06
			2	887.59	887.25	695.64	695.31	887.20	0.02	3.43	0.05
			3	628.94	628.59	572.24	571.88	628.56	0.00	3.93	0.04
			4	682.21	681.87	623.89	623.59	681.84	0.00	3.68	0.04

### Differential gene network analysis of COVID-19 severe stages

This study applied the proposed strategy to differential gene network analysis of severe stages of COVID-19. Since the proposed netRL with a Laplacian matrix constructed from regression coefficients outperformed the correlation-matrix–based approach, we employed the regression-coefficient–based netRL in this analysis. The analysis was based on whole-blood RNA-seq profiles of 1,102 genotyped individuals obtained from the Japan COVID-19 Task Force [13]. Disease severity was categorized into four stages: critical (Level 4, patients in intensive care unit or requiring intubation and ventilation, severe (Level 3, others requiring oxygen support), mild (Level 2, other symptomatic patients), and asymptomatic (Level 1, without COVID-19–related symptoms) [13]. The RNA-seq data consist of 71 asymptomatic, 241 mild, 404 severe, and 303 critical samples.

The current study aimed to identify differentially regulated molecular interplays between 303 critical and 71 asymptomatic cases. Given the imbalance in sample size between critical and asymptomatic groups, this imbalance must be carefully considered in the analysis. Therefore, this study applied the proposed netRL with the hypergeometric test to investigate differential gene networks between severe and asymptomatic COVID-19 cases. The current study focused on 1,404 genes associated with viral infectious diseases that are annotated in the “Infectious disease: viral” pathway of the Kyoto Encyclopedia of Genes and Genomes (KEGG) database (https://www.genome.jp/kegg/pathway.htm). Accordingly, all genes within this pathway, not only transcription factors(TFs), were included as candidate regulators, allowing the model to account for a broader spectrum of regulatory influences relevant to viral infection related processes, including signaling and modulatory roles carried by non-TF genes. Table 4 shows the “Infectious disease: viral”-pathways and the number of genes involved in each pathway.

**Table 4:** Infectious disease: Viral-pathways in the KEGG database.

Entry	Name	#Genes
ko05166	Human T-cell leukemia virus 1 infection	191
ko05170	Human immunodeficiency virus 1 infection	163
ko05161	Hepatitis B	132
ko05160	Hepatitis C	112
ko05171	Coronavirus disease - COVID-19	219
ko05164	Influenza A	128
ko05162	Measles	117
ko05168	Herpes simplex virus 1 infection	149
ko05163	Human cytomegalovirus infection	192
ko05167	Kaposi sarcoma-associated herpesvirus infection	169
ko05169	Epstein-Barr virus infection	172
ko05165	Human papillomavirus infection	231

The current study matched the 1404 genes and RNA-seq data from the Japan COVID-19 task force, and two gene networks of samples derived from critical and asymptomatic groups were estimated for the matched 694 genes. The total number of edges was 40,303 and 84,922 in the estimated networks of critical and asymptomatic samples, respectively.

**Fig 3:**
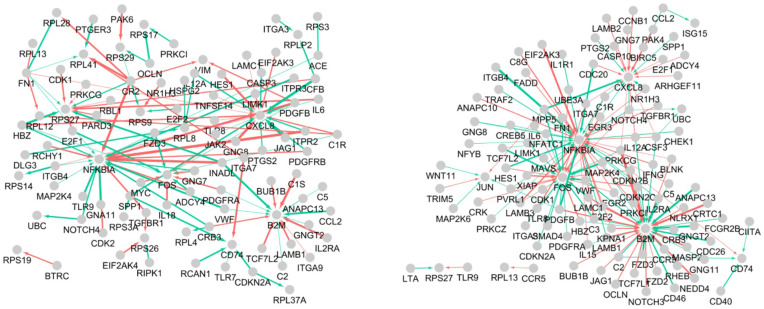
Gene regulatory networks of asymptomatic and critical COVID-19 samples. Arrows (◻→△) indicate regulatory effects from □ to △, edge thickness reflects the corresponding weight, and edge color denotes the direction of regulation (green: positive and red: negative).

Fig 3 shows the infectious disease gene regulatory networks of asymptomatic and critical COVID-19 samples. To effectively visualize the complex and large-scale gene networks, we display only the top 0.1% of edges ranked by absolute weight.

As shown in Fig 3, samples from asymptomatic samples show higher levels of molecular interactions (i.e., a large number of genes and edges) than those derived from critical individuals. The genes NFKBIA, B2M, CXCL8, and FOS emerged as hub genes in both networks. Furthermore, the interactions between ribosomal proteins (i.e., RPS and RPL genes) were identified as asymptomatic sample-specific characteristics.

- B2M
- Conca et al. [14] suggested that elevated β2-m levels may serve as an early biomarker of disease severity and could provide predictive value for the clinical trajectory and outcome of COVID-19. As noted by Zou et al. [15], abnormal expression of B2M may be an important factor underlying tracheal dysfunction induced by coronavirus infection. According to Song et al. [16], B2M was proposed as a candidate biomarker for viral myocarditis, which may provide insights on the mechanisms underlying COVID-19–related myocarditis.
- CXCL
- Pius et al. [17] proposed that CXCL8 antibody seropositivity may serve as a novel prognostic indicator for severe progression in patients with COVID-19. In severe COVID-19, CXCL8 is persistently elevated and has been proposed as a potential prognostic biomarker. Cross-viral comparisons involving SARS-CoV, MERS-CoV, and SARS-CoV-2 highlight its role as a central mediator of pulmonary pathogenesis and emphasize its relevance to COVID-19 progression [18]. Elevated levels of CXCL8 were observed in patients with severe COVID-19, suggesting a possible role in the underlying mechanisms of disease severity [19].
- FOS
- In the early recovery phase of COVID-19, CD4 + T cells exhibited pronounced upregulation of inflammatory genes, including FOS, JUN, KLF6, and S100A8, indicating their potential involvement in modulating the immune response [20]. Li et al. [21] proposed that puerarin-mediated targeting of FOS could serve as a potential therapeutic approach for clinical management of SARS-CoV-2 infection. The study by Qin et al. [22] highlighted critical molecular targets of puerarin in the context of COVID-19 treatment, encompassing novel anti–COVID-19 targets, including FOS, PTGS, PRKCB, PRKCA, and NOS3.
- NFKBIA
- Amini et al. [23] demonstrated that infection with SARS-CoV-2 leads to elevated expression of essential NF-κB signaling components, including NFKBIA, NFKB1, RELA, and NFKB2. In severe COVID-19, the NFKBIA rs696 GG genotype was associated with ICU admission. Given the central involvement of NF-κB pathway dysregulation in disease severity, this variant could represent a marker for wider COVID-19 clinical outcomes [24]. Severe COVID-19 was associated with increased neutrophil counts and higher expression of NFKBIA and TNFAIP3, whereas TNFAIP3, PPP1R15A, NFKBIA, and IFIT2 showed bimodal expression across various immune cell populations relative to that in or uninfected individuals or those with mild symptoms [25].

**Fig 4:**
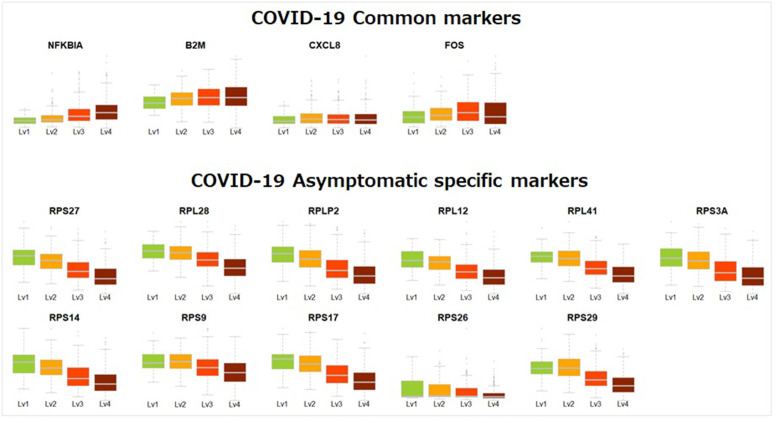
Expression levels of COVID-19 asymptomatic-specific and common markers, where Lv1: asymptomatic, Lv2: mild, Lv3: severe, Lv4: critical of COVID-19 samples.

Fig 4 shows the expression levels of the identified COVID-19 asymptomatic specific (i.e., ribosome proteins) and common (i.e., NFKBIA, B2M, CXCL8, and FOS) makers across the severity stages (Lv1: asymptomatic, Lv2: mild, Lv3: severe, Lv4: critical).

The identified markers characterize differential expression signatures corresponding to COVID-19 stages. Expression of COVID-19 common markers increased with disease progression from asymptomatic to critical stages, whereas asymptomatic-specific markers, such as ribosomal proteins, were highly expressed in asymptomatic samples and declined with disease severity. The findings demonstrate that severe COVID-19 is marked by elevated levels of the identified markers (i.e., NFKBIA, B2M, CXCL8, and FOS), in contrast to non-severe cases, which are distinguished by enhanced expression of asymptomatic-specific markers. Such molecular features may offer important clues to the mechanistic basis of disease severity.

**Fig 5:**
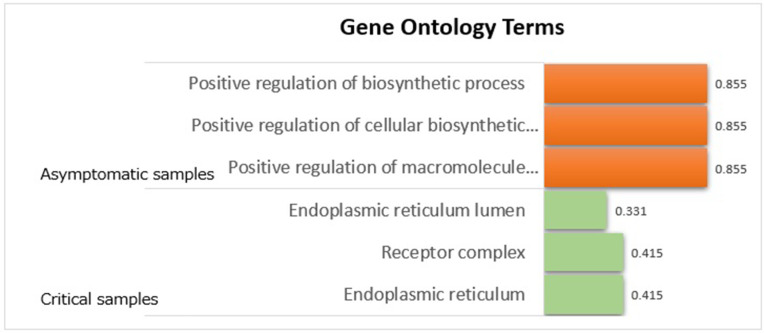
Significant Gene Ontology terms of gene networks of COVID-19 asymptomatic and critical samples.

To elucidate biological pathways and functional annotations underlying the gene networks in COVID-19 asymptomatic and critical groups, this study performed pathway analysis based on the bioinformatics tool, Database for Annotation, Visualization, and Integrated Discovery (DAVID) [26]. The genes constituting the gene networks of asymptomatic and critical samples in Fig 3 were subjected to Gene Ontology (GO) term pathway analysis, using the 1,404 viral infection disease–related genes as the background set. Fig 5 shows the top three most significant GO terms (i.e., those with the smallest FDR-adjusted q-values), where statistical significance evaluated using Benjamini–Hochberg false discovery rate (FDR)–adjusted q-values that correct for multiple testing across all GO terms.

Pathway analysis revealed that “Positive regulation”-related GO terms, i.e., (“Positive regulation of macromolecule biosynthetic process,” “Positive regulation of cellular biosynthetic process” and “Positive regulation of biosynthetic process”) were specific to COVID-19 critical samples, in contrast to “Endoplasmic”-related (i.e., “Endoplasmic reticulum,” “Endoplasmic reticulum lumen”) and “Receptor complex” GO terms were characterized the asymptomatic samples. The current study suggest that targeting the suppression of critical sample–specific GO terms (i.e., “Positive regulation”-related GO terms) may provide important insights into the underlying mechanisms of severe COVID-19.

## Discussion

This study developed network-constrained Random Lasso (netRL), a novel computational framework for gene regulatory network inference that explicitly incorporates network biological knowledge into statistical modeling. The motivation stemmed from a critical limitation in differential gene network analysis under the unequal sample sizes across phenotypes, which often leads to biased estimation and spurious phenotype-specific molecular interactions. By combining bootstrap resampling and random selection of regulators, the netRL resolved the limitation of the existing methods and achieved effective gene network inference. Furthermore, the developed strategy incorporated network biological knowledge into network estimation, and thus netRL enhances the biological interpretability and reliability of the inferred networks.

Monte Carlo simulation experiments clearly demonstrated the superiority of netRL compared with existing methods. Notably, netRL combined with the hypergeometric test yielded the most effective results across diverse simulation scenarios. While the bootstrap confidence interval–based version of netRL achieved high sensitivity, it failed to efficiently filter out false positives, particularly in settings with large numbers of potential regulators. These findings highlight that the choice of edge selection criterion plays a decisive role in determining the practical utility of network inference approaches. This study applied the netRL to uncover COVID-19 severe-specific molecular interplays. The proposed strategy successfully identified distinct gene regulatory networks for asymptomatic and critical COVID-19 samples, despite the significant sample size imbalance. The hub genes identified in the both asymptomatic critical samples (e.g., NFKBIA, B2M, CXCL8, and FOS) are consistent with those identified in existing literature on COVID-19 severity, as their expression levels are found to increase with disease progression. In contrast, asymptomatic-specific markers, such as ribosomal proteins, were highly expressed in non-severe cases, and their expression decreased with increasing severity. Moreover, pathway analysis uncovered distinct Gene Ontology terms between the asymptomatic and critical networks, further suggesting that network-based molecular signatures can provide mechanistic insights into disease progression.

In the gene network analysis of the severe stage of COVID-19, this study considered all genes within the Infectious disease: viral pathway, not only TFs, as potential regulators. While this broader inclusion allows the model to capture a wider range of regulatory influences that may contribute to viral-infection–related processes, narrowing the analysis to transcription factors could enhance the biological interpretability of the inferred network. Therefore, incorporating TF-focused regulatory modeling represents an important direction for future work in this study.

While the proposed method effectively addresses unequal sample sizes, the reliance on bootstrap resampling may increase computational burden for very large-scale genomic datasets. Future research should thus focus on developing efficient algorithms for large-scale data and on integrating multi-omics network resources to further refine inference accuracy.

Although the proposed permutation-based procedure assesses the significance of individual edges, a global multiple-testing correction across the entire network was not applied. Extending the framework to incorporate formal edge-level error control, for example through FDR control methods, is left as an important direction for future work. Furthermore, the increased computational complexity resulting of the proposed netRL from the bootstrap-based strategy may limit its applicability in extremely large-scale settings. Addressing this issue through algorithmic optimization or scalable approximations will be an important direction for future research.

## Conclusion

This study proposed network-constrained Random Lasso (netRL), a unified framework for gene network inference that explicitly addresses sample size imbalance while enhancing biological interpretability. By integrating balanced bootstrap resampling with network-informed regularization and centrality-guided subsampling, netRL provides an effective framework for high-dimensional gene network inference. Simulation studies and real-world RNA-seq analysis demonstrated that netRL effectively performed gene network inference even under severe sample imbalance. The results indicate that incorporating network structure and gene-level importance into the random lasso framework yields more robust and biologically meaningful networks than existing approaches. Overall, netRL offers a flexible and extensible platform for differential gene network analysis and can be readily applied to a wide range of complex disease studies where unequal sample sizes and high dimensionality are inherent challenges.

## Supporting information

S1 File Supplementary file of netRL.xlsx. This file provides the supplementary results of Monte Carlo Simulation.
(XLSX)
